# Gestión del proceso posanalítico en los laboratorios clínicos según los requisitos de la norma ISO 15189:2012. Consideraciones sobre la revisión, notificación y comunicación de los resultados

**DOI:** 10.1515/almed-2020-0027

**Published:** 2020-09-05

**Authors:** Ma Liboria López Yeste, Silvia Izquierdo Álvarez, Antonia R. Pons Mas, Luisa Álvarez Domínguez, Aurora Blanco Font, Fernando Marqués García, Francisco A. Bernabeu Andreu, Ma Patrocinio Chueca Rodríguez, Ana García Álvarez, Teresa Contreras Sanfeliciano, Natalia Pascual Gómez, Lorena Sánchez Gancedo, Leonor Guiñón Muñoz

**Affiliations:** CATLAB, Barcelona, España; Servicio de Bioquímica Clínica, Hospital Universitario Miguel Servet, Zaragoza, España; Servicio de Análisis Clínicos, Hospital Universitari Son Espases, Mallorca, España; Sociedad Española de Medicina de Laboratorio (SEQC^ML^), Comisión de Acreditación de Laboratorios, Barcelona, España; Laboratori Clínic, Hospital Universitari de Bellvitge, Barcelona, España; Servicio de Análisis Clínicos- Bioquímica Clínica, Hospital Universitario Puerta de Hierro, Madrid, España; Servicio Análisis Clínicos, Hospital Clínico San Carlos, Madrid, España; Servicio de Análisis Clínicos y Bioquímica Clínica, Complejo Asistencial Universitario, Salamanca, España; Servicio de Análisis Clínicos, Hospital Universitario de la Princesa, Madrid, España; Instituto de Medicina Oncológica y Molecular, Oviedo, Asturias, España; Hospital de la Santa Creu i Sant Pau, Barcelona, España

**Keywords:** acreditación, laboratorio clínico, norma ISO 15189, proceso posanalítico

## Abstract

El objeto de este trabajo es establecer unas consideraciones para facilitar la gestión del proceso posanalítico respecto a la revisión, notificación y comunicación de los resultados, de acuerdo con los requisitos de la Norma UNE-EN ISO 15189:2013. El ámbito de aplicación incluye las actividades del proceso posanalítico del laboratorio clínico, así como el personal implicado en él (dirección y personal del laboratorio). Se indican los criterios y la información necesaria para realizar la revisión y validación de los resultados de las pruebas analíticas y así enviar a los destinatarios informes claros, asegurando siempre una transcripción fidedigna de los resultados e incluyendo toda la información necesaria para su correcta interpretación. Asimismo, se describen los requisitos para una correcta comunicación de los resultados del laboratorio, haciendo especial hincapié en la comunicación de aquellos resultados alarmantes o críticos. En algunos países de Europa es obligatoria la acreditación, total o parcial, de los laboratorios clínicos, siguiendo la Norma ISO 15189 y esta parece ser la hoja de ruta marcada en otros muchos países. Por ello, es indispensable la comprensión de sus requisitos para realizar una implementación progresiva y más fácil.

## Introducción

Desde el punto de vista de la Norma UNE-EN ISO 15189:2013 (en adelante Norma ISO 15189), el proceso posanalítico se puede subdividir en dos subprocesos: uno realizado en el laboratorio, que incluye la revisión de los resultados, su introducción o traspaso al SIL (Sistema de Información del Laboratorio) y su comunicación al profesional responsable de la petición a través del informe de laboratorio [1] y un segundo que comprende actividades externas al laboratorio, donde el profesional responsable de la solicitud analítica o responsable del paciente recibe el resultado, lo interpreta y procede a la toma de decisión clínica [2].

En la actualidad, los laboratorios clínicos dedican cada vez más esfuerzo a mejorar las habilidades metodológicas y de comunicación de las actividades posanalíticas, con la finalidad de ayudar a interpretar los resultados de laboratorio, proporcionando toda la información necesaria y haciendo un especial énfasis en su significado clínico, de modo que lleven a una mejor interpretación de los resultados emitidos [3]*.* A pesar de ello, muchos resultados son inadecuadamente interpretados por el receptor y derivan en la realización de acciones equivocadas. Según la bibliografía, aproximadamente el 5% de los errores vinculados al laboratorio están relacionados con una mala interpretación del resultado, lo que provoca un 33 % de los retrasos, errores o falta de diagnóstico [3]. Otras fuentes indican que los errores en la interpretación de las pruebas analíticas (37%), junto con los errores en la solicitud (58%), son las causas más importantes de errores en el proceso diagnóstico [2], [4], [5], si bien la mayoría no llegan a producir efectos adversos sobre la salud del paciente.

Es necesario que el laboratorio aplique una metodología adecuada para la detección y la clasificación de los errores posanalíticos y adopte las herramientas apropiadas para su mitigación [6].

El objetivo y campo de aplicación de este trabajo es establecer recomendaciones para facilitar la gestión del proceso posanalítico de acuerdo con los requisitos de la Norma ISO 15189, exigidos principalmente en sus apartados 5.7, 5.8 y 5.9. En ningún caso sustituye ni amplía la Norma y ha de utilizarse como apoyo a la interpretación y aplicación de la misma. Su ámbito de aplicación son los profesionales implicados, así como las actividades comprendidas en la parte del proceso posanalítico del laboratorio clínico.

Las consideraciones indicadas en este artículo han sido desarrolladas por la Comisión de Acreditación de Laboratorios de la Sociedad Española de Medicina de Laboratorio (SEQC^ML^).

## Revisión de los resultados

El laboratorio debe documentar la sistemática establecida para la revisión de los resultados de los análisis y asegurar que la realiza personal formado y autorizado.

**Figura 1:**
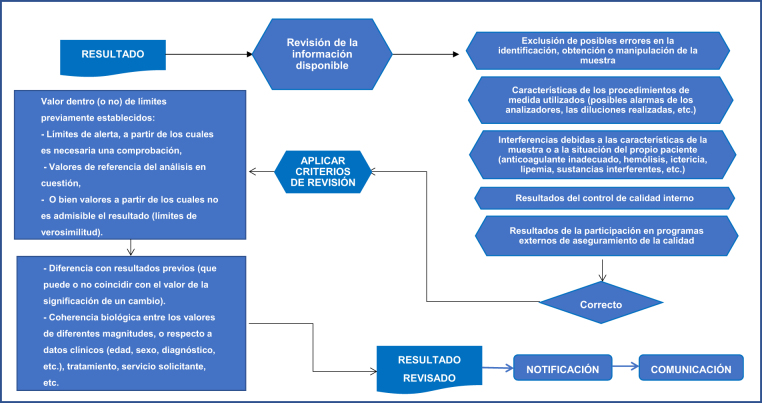
Información a considerar y criterios más utilizados en la revisión de los resultados de pacientes.

La información necesaria y los criterios utilizados más frecuentemente en la revisión de los resultados se detallan en la Figura 1.

Tras la revisión de los resultados, considerando los resultados del control de calidad interno, la información clínica disponible y los resultados previos del paciente, se realiza su emisión con la interpretación de los mismos. Es importante tener en cuenta la capacidad predictiva del análisis, pudiendo incluir la recomendación de realizar pruebas diagnósticas, sean o no de laboratorio.

En ocasiones, el laboratorio establece pruebas reflejas o concurrentes, cuya realización está condicionada por un determinado resultado en la prueba solicitada y que también se valoran durante la revisión de los resultados.

Algunos laboratorios realizan una selección y notificación automatizada de los resultados, comúnmente denominada “validación automática”, que requiere que los criterios de revisión estén bien definidos, documentados y aprobados.

En los laboratorios de urgencias, sobretodo, es frecuente la validación parcial del informe, emitiendo resultados a medida que se van obteniendo. Ello requiere una verificación añadida de la congruencia de los nuevos resultados con los anteriores.

## Notificación y edición de los resultados

Una vez revisados los resultados, deben ser enviados a los destinatarios mediante informes claros, exactos, sin ambigüedad, asegurando siempre una transcripción fidedigna de los resultados (tanto de los validados individualmente como de los validados automáticamente) e incluyendo toda la información necesaria para su correcta interpretación. Se considerarán también los requisitos establecidos en la legislación vigente, así como las recomendaciones o buenas prácticas establecidas por la comunidad científica.

Los resultados pueden ser comunicados por diferentes vías: informe en papel, informe por vía electrónica o mediante comunicación oral (siempre seguida del envío del informe escrito, ya sea en papel o electrónico). Si existen procesos de transcripción de resultados, éstos deberán estar documentados, garantizando la detección y minimización de posibles errores.

Los resultados emitidos por los laboratorios subcontratados deben identificarse como tales en el informe, asegurando su trazabilidad.

### Emisión del informe

El laboratorio debe asegurar que el informe emitido llega a la persona autorizada, al facultativo solicitante o al paciente, si se ha acordado previamente con el facultativo, garantizando la confidencialidad de los resultados.

Si el laboratorio emite informes preliminares o provisionales, deberá identificarlos claramente como tales, enviando siempre posteriormente el informe final, indicando en éste, si es posible, que sustituye al preliminar emitido en una determinada fecha.

Asimismo, el laboratorio debe definir un proceso para notificar al solicitante un retraso en la entrega de un resultado de un análisis que pueda interferir en la labor asistencial o comprometer la seguridad del paciente.

### Características del informe

En el apartado 5.8.2 de la Norma se describen las particularidades que debe cumplir el informe, de forma que garantice la comunicación de los resultados de manera efectiva y satisfaga las necesidades del solicitante, incluyendo la información de la muestra, la identificación de valores alarmantes o comentarios interpretativos, como se detalla en la Tabla 1.

**Tabla 1:** Notificación de resultados: características y contenido del informe.

Apartado del informe	Indicación
Muestra	Tipo de muestra primaria. Fecha de toma de muestra (y hora si procede, por ejemplo, en muestras para gasometrías o para estudiar niveles de fármacos). Comentarios sobre la adecuación de la muestra y la calidad de la misma cuando estas puedan afectar a los resultados de los análisis. Para ello, el laboratorio debe definir, documentar y difundir los criterios de aceptación o rechazo de las muestras y revisarlos ante cambios en el procedimiento de medida.
Análisis	El análisis (y método analítico cuando proceda).
Paciente	Identificación del paciente y ubicación (en cada página).
Solicitante	Identificación del solicitante e información de contacto.
Laboratorio	Identificación del laboratorio que lo emite. Identificación de los análisis realizados por los laboratorios subcontratados.
Resultado	Resultados y las unidades que apliquen. Intervalos de referencia o valores de decisión clínica o diagramas/nomogramas. Identificar los resultados que se encuentren en el intervalo alarmante y que puedan comprometer la seguridad del paciente y darles el tratamiento que se indica en el texto. Comentarios interpretativos de los resultados, cuando proceda. Incluir aquellos comentarios interpretativos que aporten valor a los resultados obtenidos o sean imprescindibles para su correcta interpretación. Otros comentarios (análisis realizados en el marco de una investigación o programa de desarrollo, etc.).
Revisor	Identificación de la persona que revisa y autoriza la emisión del informe.
Fechas	Fecha de solicitud. Hora de extracción de la muestra, si es un dato importante para la determinación analítica. Fecha y hora de emisión.
Paginación	Número de página y número total de páginas.
Marca	Marca y/o frase de que las determinaciones se encuentran, o no, al amparo de la acreditación.

### Contenido del informe

El informe deberá incluir la información requerida en la legislación que sea aplicable en cada país. En España, aplican las reglamentaciones de los Departamentos de Salud de cada Comunidad Autónoma, así como el Real Decreto 1093/2010, de 3 de Septiembre que, en su anexo V, detalla el conjunto mínimo de datos que deben contener los informes clínicos de resultados de pruebas de laboratorio en el Sistema Nacional de Salud (SNS) [7].

Por su parte, la Norma en el apartado 5.8.3 indica que los informes deben incluir la información relativa a la muestra, al paciente, la analítica realizada y los resultados obtenidos, como se detalla en la Tabla 1. Además, en el apartado se indica que el laboratorio debe facilitar asesoramiento clínico en la interpretación de los resultados, incluyendo la inclusión de comentarios interpretativos [1].

Cuando proceda, se deberán incorporar comentarios interpretativos de los resultados, lo que requiere de un personal cualificado, ya que la información o comentarios erróneos podrían comprometer las conclusiones del receptor del informe y dar lugar a errores en la actividad asistencial [8], [9]. En las ocasiones en que se incluyan comentarios a resultados de pruebas realizadas en un laboratorio subcontratado, debe quedar claro en el informe quién realiza el comentario.

La inclusión o no de los comentarios dependerá́ de la disponibilidad de los datos clínicos (contexto en el cual se solicitó el análisis o factores del paciente que pueden influir en los resultados como la medicación, etc.), de la necesidad de emprender acciones inmediatas, de la experiencia del solicitante con ese análisis y su interpretación, o de que los resultados obtenidos no sean los esperados. En ocasiones, es necesario incluir información sobre la limitación de los métodos, por ejemplo, en los informes de pruebas genéticas.

El laboratorio también debe valorar la conveniencia de incluir comentarios interpretativos en los resultados de un determinado análisis cuando así lo soliciten los facultativos a quienes va dirigido, y cuando se haya consensuado con ellos. Por tanto, el tipo de comentario interpretativo dependerá́ de la complejidad de la prueba analítica, del servicio clínico peticionario y de la capacidad del destinatario para la interpretación del comentario. La falta de armonización en cuanto a metodología, unidades de medida, intervalos de referencia o límites de decisión y la introducción de nuevas y complejas pruebas de laboratorio, hacen que el asesoramiento proporcionado con los comentarios sea especialmente valorado por quién recibe el informe [10].

Se deben evitar comentarios que no aporten valor a la interpretación del resultado (como, por ejemplo, que un valor es elevado junto al intervalo de referencia), o reafirmar una cuestión clínica ya conocida, por ejemplo, en una solicitud con diagnóstico de hipotiroidismo añadir el comentario “considerar hipotiroidismo”.

El comentario ideal sería aquel que describe la normalidad/anormalidad de un resultado de medida con una interpretación de la información que aporta conocimiento para el seguimiento, recomendando, si aplica, pruebas adicionales o una derivación al especialista [10], [11], [12].

Estos comentarios interpretativos deben estar documentados, además de estar consensuados y estandarizados por el personal facultativo del laboratorio autorizado para la revisión de los resultados.

## Comunicación y entrega de los resultados

**Figura 2:**
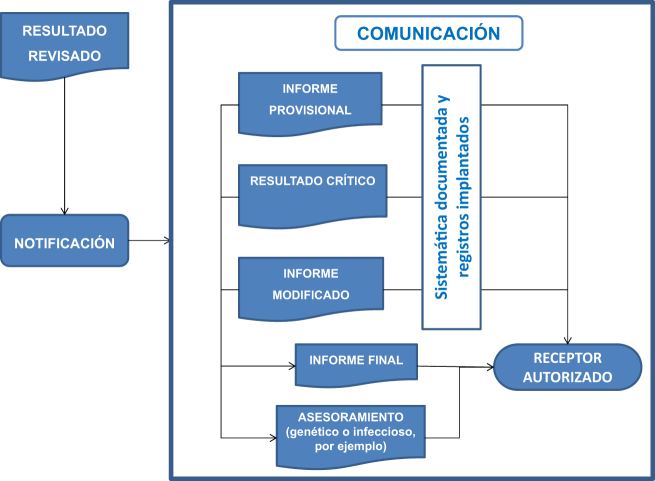
Condiciones a tener en cuenta en la comunicación de los resultados.

La Norma ISO 15189 describe la necesidad de que cada laboratorio disponga de procedimientos documentados para la comunicación de los resultados, en los que se incluya quién comunica y a quién deben comunicarse. En su apartado 5.9 especifica las condiciones a tener en cuenta en esta comunicación (ver Figura 2).

El informe del laboratorio puede ser generado directamente por el SIL o bien que el SIL realice el envío de los resultados a otros sistemas de información de ámbito hospitalario o comunitario, para que éstos lo generen. En caso de comunicarse resultados oralmente, debe llevarse a cabo un registro de los mismos para asegurar su trazabilidad (quién, a quién, el qué, cuándo, etc.) y deben ir seguidos siempre de un informe de laboratorio escrito.

El laboratorio debe asegurarse de que los resultados de los análisis, la información asociada y los comentarios quedan integrados con el resto de la información clínica y que se reproducen de forma exacta, tanto en formato electrónico como en papel, por los sistemas de información externos al laboratorio previstos para recibir directamente la información, teniendo en cuenta el cumplimiento de la legislación en protección de datos (si se utiliza correo electrónico, por ejemplo). Del mismo modo, si se incorpora un análisis o un comentario automatizado nuevo, se debe comprobar que estas actualizaciones se reproducen de forma exacta por los sistemas de información externos al laboratorio (impresión en papel, visualización en pantalla, etc.).

Si se produce un cambio del intervalo de referencia biológico de alguna de las pruebas analíticas, se ha de comunicar a los facultativos peticionarios, por ejemplo añadiendo un comentario en el informe, durante un período de tiempo determinado, que indique esa circunstancia.

### Comunicación de los resultados alarmantes

Desde que, en el año 1972, Lundberg describió el concepto de “valor crítico” [13], la comunicación de resultados alarmantes se ha implementado ampliamente en los laboratorios clínicos. Es responsabilidad del laboratorio protocolizar su correcta identificación y rápida comunicación, contribuyendo así al cuidado y la preservación de la seguridad del paciente, siendo además un proceso obligado en el contexto de la asistencia orientada al paciente [14]*.*

La Norma ISO 15189 incluye como requisito disponer de procedimientos documentados para informar estos resultados. La llegada oportuna del informe, junto con la confirmación de la recepción de la información, permitirá reducir los efectos adversos derivados de la demora o no comunicación de dichos resultados [15]. Además del compromiso asistencial, se debe tener en cuenta que la no comunicación de estos resultados puede constituir una infracción sanitaria grave, de acuerdo a lo que se indica, por ejemplo, en la Ley General de Sanidad española [16].

Cuando se detecta un resultado alarmante y antes de su comunicación al solicitante, el laboratorio debe comprobar que el proceso analítico se ha desarrollado correctamente (calibración, resultados de los controles, diluciones, etc.), revisar si el paciente tiene unos valores críticos anteriores ya comunicados, si hay o no diferencias significativas con dichos resultados anteriores, si el diagnóstico es conocido o si los resultados anteriores son compatibles con el valor crítico detectado. Se debe comprobar, asimismo, que no existan errores de identificación de la muestra (si se ha utilizado una alícuota no obtenida de manera automatizada es mejor verificar el resultado en el tubo primario) y si ésta es aceptable para considerar válido el resultado (presencia o no de hemólisis, lipemia, etc.) [17]. El laboratorio puede tener definidos y documentados en sus procedimientos, criterios por los que no debe ser avisado un resultado que ha definido como crítico; por ejemplo, para algunas determinaciones el criterio puede ser tener un resultado previo en menos de 24 horas en un paciente hospitalizado. En estos casos, se recomienda registrar el motivo de tal decisión (resultados previos, situación clínica del paciente, acuerdo con un servicio en particular, etc.).

Ahora bien, el tema de armonizar cuáles han de ser los límites para los resultados críticos es controvertido. El Colegio Americano de Patólogos (CAP) realizó el estudio “*Q-Probes study* 2002” [18], en el que no consiguió establecer por consenso una lista nacional estándar de resultados críticos, pero propuso una lista genérica que sirviera de punto de partida para que cada laboratorio desarrollase la suya. El responsable del laboratorio debe ser el encargado de elaborar y aprobar un listado de resultados críticos, consensuado con los responsables clínicos y teniendo en cuenta la población a la que atiende, la prevalencia de las enfermedades atendidas, las especialidades o los programas especiales existentes y estableciendo el procedimiento de comunicación según las características particulares de su centro [19]. También deben registrarse los resultados críticos comunicados y los datos relacionados con la comunicación (quién, a quién, cuándo y cómo registrarlo). La información que debe registrarse se detalla en la Tabla 2. Además, se debe evitar la comunicación innecesaria de los resultados y priorizar la comunicación de resultados con una mayor repercusión potencial inmediata sobre la vida o la salud del paciente, así como documentar la gestión que se hará ante la imposibilidad de comunicación con el solicitante de la analítica.

**Tabla 2:** Datos relacionados con los valores alarmantes informados.

Datos relacionados con los valores alarmantes informados
Identificación del paciente.
Resultado de la prueba.
Tipo de muestra.
Fecha y hora de la revisión del resultado.
Fecha y hora de la notificación.
Identificación de la persona que comunica el resultado
Identificación de la persona que recibe la comunicación.
Medio por el cual se realiza la comunicación.
Confirmación de la recepción del resultado o, en el caso de que no se haya podido efectuar la comunicación dentro del periodo de tiempo establecido en el protocolo, descripción del motivo.
**Recomendaciones**
Se deben documentar todas las notificaciones, incluyendo los intentos fallidos de comunicación de un resultado alarmante.
Cuando sea útil, registrar la comunicación de los resultados alarmantes en el propio informe de resultados.
Cuando sea posible, utilizar el SIL y su conexión con el SIH para la comunicación de los resultados críticos, debido a la trazabilidad que ofrecen, la amplia accesibilidad desde cualquier ubicación y la rapidez de comunicación.
La comunicación electrónica debe limitarse a los correos electrónicos institucionales o corporativos, de forma que se asegure la recepción por personal autorizado y el cumplimiento de la confidencialidad de la información comunicada.

Es especialmente importante el consenso con los facultativos clínicos a quienes va destinado el aviso de un resultado alarmante. Por ejemplo, el mismo resultado para la concentración de substancia de ión potasio en suero no se valora de igual manera por un nefrólogo, un intensivista o un facultativo de atención primaria, porque sus pacientes tienen enfermedades diferentes y la asistencia se lleva a cabo en un contexto asistencial diferente. En este sentido, se han hecho estudios que utilizan el consenso entre el laboratorio y los médicos de diferentes especialidades para proponer un conjunto de límites comunes para los valores de alarma, llegando asimismo a diversas conclusiones sobre el medio de comunicación de estos valores y su destinatario preferente [20], [21], [22]. También se debe definir y documentar el orden de preferencia respecto a quién comunicar el resultado crítico (facultativo peticionario, facultativo de guardia, personal de enfermería, médicos de Atención Primaria, servicio 061, etc.). En caso de comunicación al paciente o familiar responsable, siempre consensuada previamente con los clínicos y sólo en casos muy concretos, por imposibilidad de contacto con algún profesional (después del periodo de tiempo límite establecido por el laboratorio para informar de un valor crítico), es aconsejable que el personal de laboratorio le recomiende que acuda al centro sanitario capaz de proporcionar asesoramiento y tratamiento urgente adecuado.

En ocasiones, la sistemática establecida en el laboratorio para la comunicación de estos resultados se consensua con su sistema de salud. Así, entre las directrices dadas por el SNS, a través del documento "Estrategia de Seguridad del paciente 2015-2020" está la “promoción de la comunicación entre los profesionales, para lo que se han de utilizar técnicas de comunicación estructurada y se han de establecer acciones para la comunicación efectiva y a tiempo de los valores de alerta, alarma o críticos que pueden poner en peligro la vida del paciente” [23].

**Figura 3:**
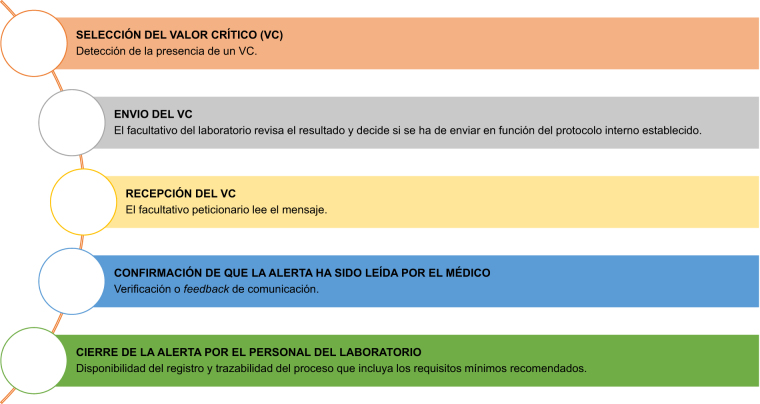
Secuencia de acciones que integran el envío de un valor crítico.

En la Figura 3 se detalla la secuencia de acciones que intervienen en la comunicación de los resultados alarmantes.

### Selección y notificación automatizada de los resultados

Muchos de los analizadores actuales permiten realizar una primera validación de los resultados obtenidos. El facultativo responsable decide los criterios de esta validación automatizada, que deben estar documentados y ser específicos de cada magnitud. Estos criterios pueden ser los límites de referencia (ajustados por edad, sexo u otras condiciones del paciente) o un múltiplo de éstos, los límites para establecer valores alarmantes, el *delta check* (cambio de valor respecto a otro anterior en un periodo de tiempo dado), relaciones matemáticas entre resultados de pruebas relacionadas entre sí, el diagnóstico o la procedencia del paciente, etc. También pueden establecerse como criterio los límites de validación que dejan estadísticamente una proporción de resultados para su revisión individual. El facultativo los utiliza de varias formas, a través de la gestión de resultados de los programas que controlan el funcionamiento de los propios analizadores, mediante el SIL al cual transmiten los resultados, o bien mediante un aplicativo *middleware* intermedio entre ambos. En cualquier caso, los resultados validados automáticamente corresponden al intervalo de valores que no implican una alteración relevante o patología clínica y no necesitan una validación facultativa de manera individual.

El SIL, por tanto, ha de permitir disponer de filtros que identifiquen las peticiones de un determinado origen o aquellas que presenten resultados con determinados valores. Son recomendables los sistemas expertos que, mediante algoritmos, permitan identificar los resultados que necesitan una revisión del facultativo, por ser incongruentes o necesitar comentarios o recomendaciones adicionales.

### Informes de laboratorio corregidos

El laboratorio debe documentar las instrucciones sobre la corrección de los informes. Cuando se realice alguna corrección se debe añadir un comentario en el informe, explicando el motivo de la modificación de forma clara y concisa, indicando la fecha de la modificación (la hora debe estar accesible en el laboratorio) así como la identificación de la persona responsable de la misma. Estos resultados modificados se deben conservar en los informes acumulativos subsiguientes, así como las anotaciones originales (no se pueden eliminar del SIL). Siempre se debe comunicar al facultativo solicitante la corrección realizada, ya que podría dar lugar a un cambio en el diagnóstico, tratamiento o seguimiento del paciente.

### Notificación de los resultados de laboratorios externos

La Norma, en el punto 4.5.2, refleja la posibilidad de provisión de los resultados de análisis de los laboratorios y consultores subcontratistas y aclara que es el laboratorio solicitante el responsable de asegurar que los resultados llegan a la persona que efectúa la petición.

Se debe disponer de un procedimiento documentado en el que se indiquen los laboratorios subcontratados, así como los acuerdos y las revisiones periódicas de cumplimiento de los criterios de selección y control. Debe existir, asimismo, un registro de las peticiones, las muestras y los resultados de todos los análisis subcontratados, de forma que permita la trazabilidad del proceso.

Aunque se debe evitar en lo posible, en aquellos casos en que es necesaria la transcripción de los resultados, por la ausencia o dificultad de comunicación entre los SIL de ambas partes, el laboratorio ha de asegurar que la transcripción es fidedigna, mediante la incorporación de los informes externos en formato electrónico (por ejemplo PDF) a las peticiones electrónicas solicitadas. También, el laboratorio debe asegurar la identificación inequívoca en la incorporación de estos informes en el SIL y establecer un procedimiento escrito para verificar el cumplimiento del circuito.

La normalización de las actividades en los laboratorios externos aseguraría una sistemática de trabajo sobre la que sería más sencillo integrar un sistema de gestión de la calidad en actividades compartidas entre centros diferentes y con SIL distintos. Son pocos los documentos que intentan abordar la normalización de la subcontratación de las pruebas enviadas a laboratorios externos [24], [25]*.*

La revisión facultativa de los resultados de las pruebas realizadas en laboratorios subcontratados suele realizarse por el laboratorio externo que realiza la prueba, en quién recae toda la responsabilidad del resultado. Por otro lado, el laboratorio debe asegurarse de que el centro subcontratado cumple los requisitos preestablecidos en la subcontratación (información necesaria para la obtención de la muestra, de conservación, envío, términos para la entrega de resultados, contenido del informe, coste, o cualquier otro requisito relevante), así como que haya utilizado un procedimiento de medida validado, que dispone de un plan de control interno de la calidad analítica y participa en programas de intercomparación. Puede además, revisar los valores de referencia y concertar quién emite los resultados y, en el informe, ha de quedar claro que el análisis se ha realizado en el centro externo concreto. Si los resultados de los análisis subcontratados incluyen valores críticos, debe establecerse el circuito y el responsable de su comunicación.

Cuando las unidades o valores de referencia sean diferentes a las del archivo histórico del paciente, estos cambios se deben notificar al facultativo solicitante en la forma y formato que el laboratorio considere más útil para la correcta difusión de dicha información.

En algunos casos, puede subcontratarse únicamente una parte del procedimiento analítico: la revisión facultativa e interpretación de los resultados de estas pruebas puede también subcontratarse o ser realizada por el laboratorio solicitante, aunque siempre es éste el responsable de asegurar la correcta comunicación del resultado.

En la Tabla 3 se resumen los requisitos y registros requeridos para la revisión, notificación y comunicación de los resultados del laboratorio, según la Norma UNE-EN ISO 15189:2013.

**Tabla 3:** Resumen de los requisitos y registros requeridos para la revisión, notificación y comunicación de los resultados del laboratorio, según la Norma UNE-EN ISO 15189:2013.

Aspectos a documentar	Requisitos a establecer en el procedimiento	Registros (en el SIL o en otro soporte)
Revisión de los resultados	Control de calidad interno correcto. Concordancia con información clínica. Concordancia con resultados anteriores (definir la desviación permitida). Criterios que determinan la repetición. Alertas y/o intervalos definidos en cuanto a: Emisión automática. Revisión facultativa. Valores críticos.	Resultados calibraciones y controles. Resultados de las analíticas. Persona que revisa/valida los resultados. Si la emisión ha sido o no automática. Resultado repetido y original. Valores críticos comunicados, persona que comunica, fecha, hora y persona que lo recibe.
Notificación de los resultados	Informe electrónico o en papel. Formato (ver contenido en el texto). Forma de comunicación. Expresión del resultado (unidades, comentarios si aplica, valores de referencia al lado, etc.). Manera de notificar un retraso. Describir la vía de aseguramiento de que el solicitante conoce la modificación.	Cambios en la manera de notificar los resultados, fecha y persona que los hace. Cambios en la expresión del resultado, fecha y persona que los hace. Notificaciones de retrasos y persona que lo hace. Modificaciones del formato del informe realizadas, que incluya día, hora y persona que las autoriza.
Comunicación de los resultados	Circuitos de comunicación. Conexiones de comunicación. Manera de controlar las transcripciones. Parciales y finales. Sistemática de comunicación por teléfono o correo electrónico. Sistemática de comunicación de resultados especiales. Sistemática de interrupción y de notificación de resultados incorrectos.	Persona que comunica los resultados. Personas de contacto, que reciben la comunicación. Controles de las transcripciones. Resultados facilitados por teléfono o correo electrónico. Incidencias/no conformidades detectadas por fallos en la comunicación o resultados incorrectos.
Selección y notificación automatizadas de los resultados	Criterios de selección y notificación automatizadas.	Validación de los criterios de selección y notificación automatizadas. Fecha y hora de la selección y notificación.
Informes de laboratorio corregidos	Instrucciones para la corrección de un informe original. Determinar cómo asegurarse de que el solicitante conoce una corrección.	Modificaciones realizadas incluyendo día, hora y persona que las autoriza, conservando los datos originales. Evidencia de que el solicitante conoce la corrección de un informe.
